# Supplementary material for: The Recovery of Epidermal Proliferation Pattern in Human Skin Xenograft
Source: Cells. 2025 Mar 17;14(6):448. doi: 10.3390/cells14060448 (PMC11941497; doi:10.3390/cells14060448)
Supplement: Supplementary file 1 [file cells-14-00448-s001.zip › cells-3359416-supplementary.pdf]

Appendix A. Distribution of Ki67+/BrdU+ Cell Density

The QuPath software was utilized for the detection of BrdU and Ki67 in epidermal keratinocyte nuclei on IHC-stained cryosections of xenografts. A classification was assigned to each nucleus based on the presence or absence of staining (negative, Ki67 positive, BrdU positive, or double-positive) (Figure 1a).

The spatial distribution of regions exhibiting a higher density of Ki67+/BrdU+ cells was investigated for identification of specific locations within the epidermis that are associated with elevated proliferation level. Regions with high densities of BrdU/Ki67-positive nuclei were referred to as "hotspots". The majority of hotspots were situated within the rete ridges (Figure S1a-c), indicating a correlation between level of proliferation and these regions. However, no associations were identified with regard to HFs in head xenografts. Hotspots were observed to be distributed throughout the epidermis (Figure S1b,c).

To verify the correlation between epidermal rete ridges and proliferative cell density, a parameter termed "epidermal thickness" was introduced. It was calculated as the sum of distances to the basement membrane and to the epidermal surface for each nucleus; thereby, nuclei situated in regions of greater epidermal thickness were more likely to be located in rete ridges.

The epidermal thickness for Ki67+/BrdU- and Ki67+/BrdU+ cells in the head, and for Ki67+/BrdU- cells in abdomen, was found to be greater than that of nuclei negative for Ki67 (BrdU+/-) (Figure S1d, e), indicating the crowding of Ki67-positive nuclei within the rete ridges. Concurrently, a comparable analysis did not reveal notable modifications in the proliferation pattern for all nuclei categories in relation to their distance from the HFs (Figure S1f).

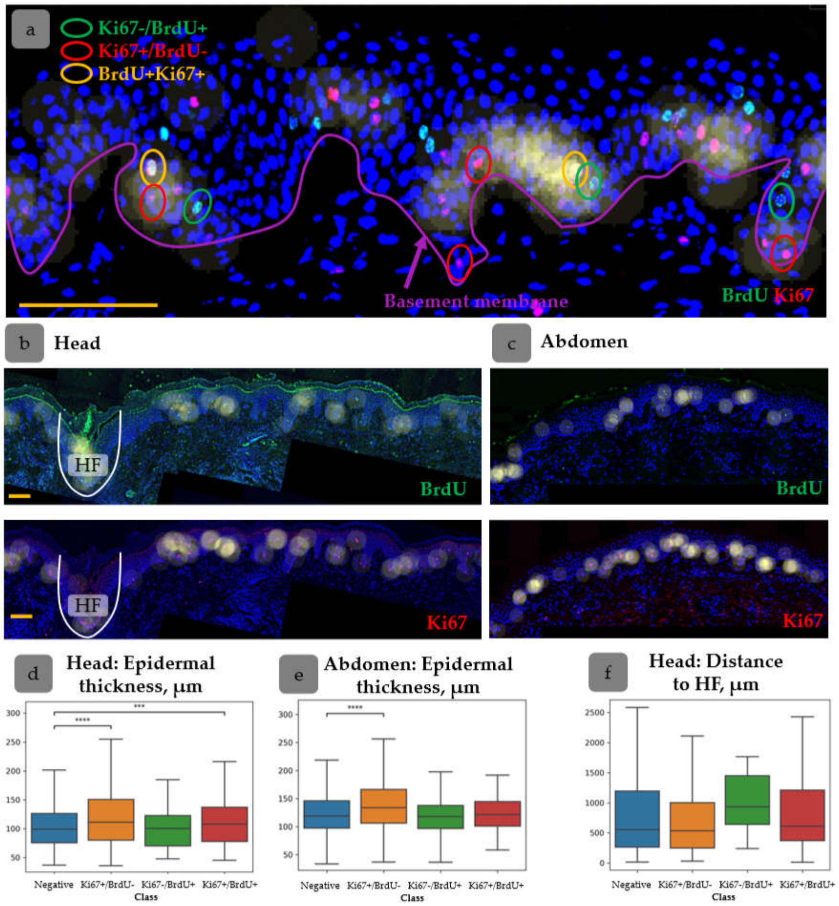

Figure S1. (a-c) Density maps of cells positive for Ki67 (a, c) or BrdU (b). Examples of Ki67-/BrdU+, Ki67+/BrdU-, and BrdU+Ki67+ cells in (a) are marked by colored circles. Yellow spots – places with higher densities of positive nuclei. (b, c) Representative density maps of proliferating epidermal cells in head xenografts with the HFs in the observation field (b) and abdominal (c) xenografts. Blue channel – DAPI. Scale bar: 100 µm. (d, e) Mean epidermal thickness for each nuclei class in head (d) and abdominal (e) xenografts. (f) Mean distance to HF for each nuclei class. *** - $p<0,001$; **** - $p<0,0001$ (one-way ANOVA with Tukey correction for multiple comparisons).

Appendix B. Parameters for Cell Clustering

Appendix B.1. Parameters for Cell Clustering: BrdU Intensity

In order to cluster the nuclei using machine learning instruments, we selected and analyzed several parameters. BrdU intensity provided insight into the extent of cell division following the pulse, whereas Ki67 staining was contingent upon the cell cycle stage. The fluorescence intensities in the channels for BrdU and Ki67 staining were measured for each nucleus, thereby providing information about the proliferative status of the cells.

The nuclei were divided into four groups based on BrdU staining intensity: low BrdU level indicated frequently dividing cells; medium BrdU level was indicated cells with medium frequency of divisions; high BrdU level represented rarely dividing cells; and the last group included BrdU-negative nuclei (Figure S2a). Analyzing Ki67 staining in groups, it was identified that Ki67 intensity increased in the same manner as BrdU in head skin xenografts (Figure S2b). The trend only deviated for the group with maximum BrdU intensity that included cells with the highest Ki67 expression as well as differentiated Ki67 negative cells (Figure S2a,b). Epidermal thickness was found to be greater for cells exhibiting frequent division compared to cells displaying medium and low division rates. This suggested that the former were predominantly located in rete ridges. (Figure S2c).

However, no significant differences in Ki67 intensity or epidermal thickness were observed for the groups with different BrdU intensities in abdominal skin (Figure S2e,f). These alterations may represent the difference in proliferation patterns between abdominal and head regions.

The distribution of cells with rare and medium division levels was not affected by HF position in the head xenograft. Conversely, rarely dividing cells were found to be concentrated in remote locations from HFs. (Figure S2d). This finding supported the correlation between proliferation level and epidermal relief.

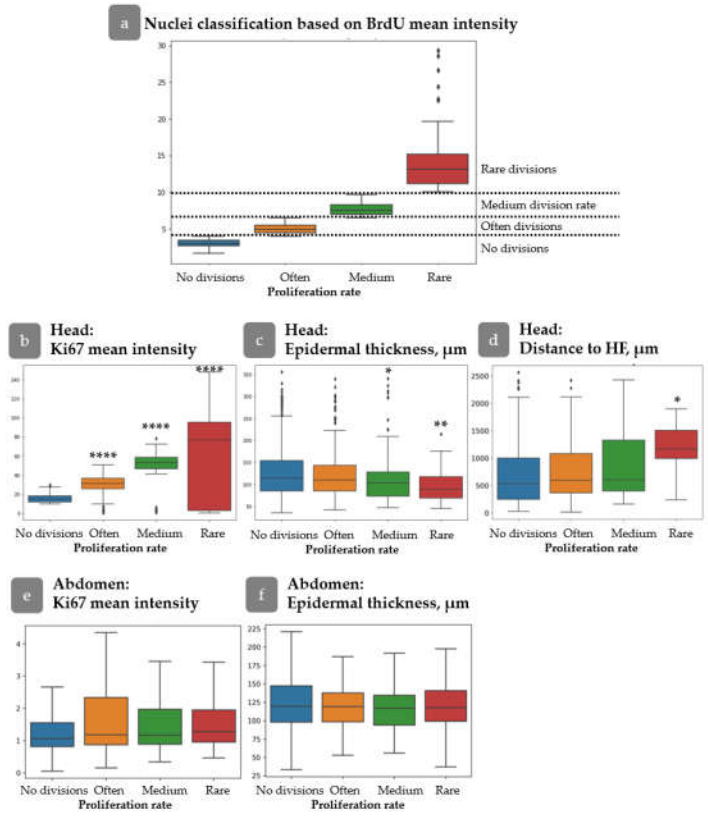

Figure S2. (a) Nuclei class assignment based on BrdU fluorescence intensity. The nuclei were divided into four groups based on BrdU fluorescence intensity. (b, e) Ki67 mean intensity at each identified class in the head xenograft shows high correlation with BrdU intensity, (b) while in abdominal (e) xenograft no correlation is observed. (c, f) Epidermal thickness in the head (c) and abdominal (f) xenograft (d) Distance to HF in the head xenograft. * - p<0,05; ** - p<0,01; **** - p<0,0001 relative to the group with no divisions (one-way ANOVA with Tukey correction for multiple comparisons).

Appendix B.2. Parameters for Cell Clustering: Nuclear Shape

The alterations in nuclear morphology that occur following the onset of keratinocyte differentiation have been previously described: keratinocytes located in the basal epidermal layer exhibit vertically oriented ellipsoid nuclei and acquire a horizontally oriented spheroid form during the process of terminal differentiation [35,36]. Nuclear morphology can also reflect their proliferation; the larger area of actively proliferating Ki67+ nuclei has been shown in mice [35]. Therefore, the parameters of nuclear shape could be a suitable tool to detect populations with different proliferation capacities. We measured the nuclear area and circularity. Nucleus circularity was calculated as the ratio between the minimal diameter and the maximal diameter of a given object. A circularity value of 1 indicates a perfectly round nucleus, whereas a lower value indicates an increasingly elongated shape.

Firstly, we confirmed that nuclei of basal cells (identified by the distance from basement membrane <10 μm) are more elongated than in suprabasal layers. The mean nuclear circularity for the basal layer was 0,80±0,10, while for upper layers it was 0,82±0,09. Basal nuclei were also slightly smaller compared to those in the suprabasal layers (Figure S3a,b,g,h,n,m).

We studied whether nuclear area and circularity are related to Ki67 and BrdU fluorescence intensities. Nuclei positive for Ki67 had a larger area, and their shape was closer to circular, while BrdU+/Ki67- cells did not differ in shape from the negative nuclei (Figure S3c,d). The grouping of cells according to their BrdU intensity revealed that cells with frequent divisions and a medium division rate exhibited a larger area and a more circular shape than cells with a low rate or that had undergone no divisions. (Figure S3e, f). The observed patterns of nuclear morphology in abdominal skin resembled those in the head region; however, differences in circularity between the studied groups were less pronounced (Figure S3i-l). Given that nuclear shape offers supplementary insight into proliferation and stemness status, it was incorporated as a variable in our cluster analysis.

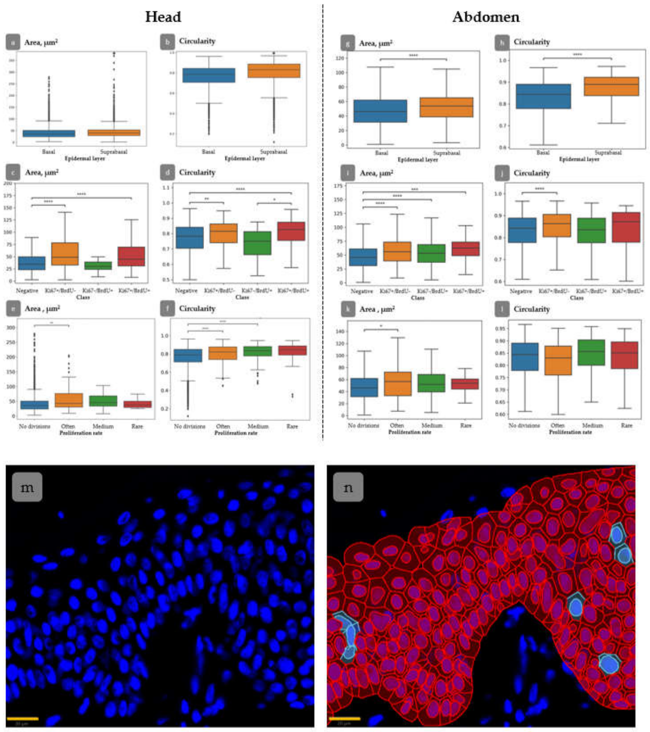

Figure S3. Morphology of nuclei with different Ki67 and BrdU statuses. Nuclear area (head—a, c, e, abdomen—g, i k) and circularity (head—b, d, f, abdomen—h, j, l) are shown for basal cells compared to the cells from suprabasal layers (a, b, g, h); for Negative, Ki67+/BrdU-, Ki67-/BrdU+ or Ki67+/BrdU+ cells. (c, d, i, j,) and for cells with different proliferation rates measured by BrdU intensity (e, f, k, l). * - p<0,05; ** - p<0,01; **** - p<0,0001 (one-way ANOVA with Tukey correction for multiple comparisons). (n, m) Images representing the morphology of nuclei. (n) IHC image of DAPI-stained skin. (m) Cells detected with the QuPath software overlaid onto DAPI. The elongated nuclei in the basal epidermal layer may be visually identified. Scale bar—20 μm.
